# Quantitative Epistasis Analysis and Pathway Inference from Genetic Interaction Data

**DOI:** 10.1371/journal.pcbi.1002048

**Published:** 2011-05-12

**Authors:** Hilary Phenix, Katy Morin, Cory Batenchuk, Jacob Parker, Vida Abedi, Liu Yang, Lioudmila Tepliakova, Theodore J. Perkins, Mads Kærn

**Affiliations:** 1 Ottawa Institute of Systems Biology, University of Ottawa, Ottawa, Ontario, Canada; 2 Department of Cellular and Molecular Medicine, University of Ottawa, Ottawa, Ontario, Canada; 3 Department of Biochemistry, Immunology and Microbiology, University of Ottawa, Ottawa, Ontario, Canada; 4 Ottawa Hospital Research Institute, Ottawa, Ontario, Canada; 5 Department of Applied Mathematics, University of Waterloo, Waterloo, Ontario, Canada; 6 Department of Physics, University of Ottawa, Ottawa, Ontario, Canada; Duke University, United States of America

## Abstract

Inferring regulatory and metabolic network models from quantitative genetic interaction data remains a major challenge in systems biology. Here, we present a novel quantitative model for interpreting epistasis within pathways responding to an external signal. The model provides the basis of an experimental method to determine the architecture of such pathways, and establishes a new set of rules to infer the order of genes within them. The method also allows the extraction of quantitative parameters enabling a new level of information to be added to genetic network models. It is applicable to any system where the impact of combinatorial loss-of-function mutations can be quantified with sufficient accuracy. We test the method by conducting a systematic analysis of a thoroughly characterized eukaryotic gene network, the galactose utilization pathway in *Saccharomyces cerevisiae*. For this purpose, we quantify the effects of single and double gene deletions on two phenotypic traits, fitness and reporter gene expression. We show that applying our method to fitness traits reveals the order of metabolic enzymes and the effects of accumulating metabolic intermediates. Conversely, the analysis of expression traits reveals the order of transcriptional regulatory genes, secondary regulatory signals and their relative strength. Strikingly, when the analyses of the two traits are combined, the method correctly infers ∼80% of the known relationships without any false positives.

## Author Summary

Cells have evolved elaborate pathways that allow them to optimally use available nutrients, for example, and alter gene expression in response to external challenges. The mapping of these pathways provides an understanding of cell function critical for advancements in a number of fields, from biofuel production to drug discovery. In this study, we developed a novel method to map pathways of genes that function in the cellular response to a given signal or stress. The method represents a significant advancement since it takes full advantage of modern genomics techniques to provide novel, detailed information about gene function, including the contribution from different genes individually, and in combination with other genes or pathways. We tested the method on a pathway in yeast whose human equivalent is associated with a serious and potentially fatal hereditary disease called galactosemia. We demonstrate that the method allows a highly accurate reconstruction of this pathway, correctly segregating genes with major and minor functions, and recapitulating the known mechanisms associated with the disease.

## Introduction

Inferring biological pathways and gene networks from measurements of genotype-phenotype relationships has been a central problem in genetics research for decades [1], [2]. These relationships represent the basic building blocks of biological network models, and underpin much of our knowledge about genes and their functions. The problem is not fully solved despite tremendous progress in the development of tools and resources enabling quantitative measurements of genetic interactions [3]–[5]. This is in part because of the uncertain biological basis of complex traits [6], and in part because methods used to analyze genetic interaction data rarely take advantage of its quantitative nature [7].

We have developed a method to infer and quantify causal relationships within hierarchical pathways responding to an external signal from genetic interaction data. Avery and Wasserman addressed part of the inference problem [8] by examining the hypothetical effects of single and double loss-of-function or constitutive mutations in the presence and absence of the signal. Based on the assumption that the signal and the two genes are either ON or OFF, with no intermediate levels of activity, they deduced a set of rules to infer which gene acts upstream of the other and whether it activates or represses the downstream gene. When applied to gene deletions, these rules can be stated as follows:

1. A given gene deletion must impact the trait when the signal is ON, or when the signal is OFF, but not both.
2. If two gene deletions impact the trait in opposite signal states, and one masks the impact of the other, then the masked gene is upstream and represses the downstream gene.
3. If two gene deletions impact the trait in the same signal state, and one masks the impact of the other, then the masked gene is downstream and is activated by the upstream gene.

The requirement of masking restricts the inference to a special subclass of genetic interactions commonly referred to as epistatic interactions. While epistasis is sometimes used synonymously with genetic interactions in general (see [9], [10] for discussion), we use the term in reference to an interaction where the mutation of one gene masks the impact of mutating another. Correspondingly, we refer to the identification of masking gene pairs, and the inference of order and causality among them, as epistasis analysis.

The Avery-Wasserman rules, which have been used broadly to interpret epistasis, suffer several shortcomings. For one, many genes have both signal-independent and signal-specific functions. The Avery-Wasserman rules are not applicable in this case since gene deletion will have an impact when the signal is OFF and when it is ON. Additionally, the rules offer no means to quantify the relative contributions of different pathways on a trait, or to assign weights to different influences within a given pathway. Therefore, important information is lost when the rules are applied to quantitative data.

To address these limitations, we develop and benchmark a novel method for epistasis analysis. The method takes full advantage of quantitative trait measurements, and enables pathway inference even when the Avery-Wasserman rules cannot be applied. We developed the method using a theoretical model incorporating signal-independent and signal-specific gene function, as well as feedforward loops. These loops allow the signal to influence the trait independently of the two genes, and the upstream gene to influence the trait independently of the downstream gene. We show that the Avery-Wasserman rules correspond to special instances of the model, and determine the assumptions required for them to be valid. Additionally, we use the model to derive a unique rule for inferring gene order in signal-responsive pathways.

Our inference method involves comparing the effects of combinatorial gene deletions measured experimentally to those predicted by our model for different hypothetical pathways. In this aspect, it is related to a ‘best-fit’ model discrimination approach to analyze quantitative genetic interaction data [11]. However, our method differs in a number of important ways: (i) we incorporate the external signal into a single model, (ii) we retain the notion that epistasis can be observed even when the assumptions made by Avery and Wasserman are invalid, (iii) we use both the signs and magnitudes of gene deletion effects to enable complete pathway inference, and (iv) we require that the experimental data be fully consistent with a hypothetical pathway that predicts epistasis. Correspondingly, our method seeks to interpret only the subset of genetic interactions for which masking is observed.

We demonstrate that our method, in addition to pathway inference, enables quantification of influences describing the function of the inferred pathway as a whole. This is an appealing feature that can be used to answer important biological questions: How much of the effect of the signal is mediated through the two genes? To what degree does the upstream gene affect the trait directly? How much of the effect is mediated through the downstream gene? These questions cannot be answered by currently available methods for epistasis analysis.

We assess the strengths and limitations of our method by analyzing genetic interaction data generated for all gene pairs in the yeast galactose utilization pathway. We chose this particular system because it is thoroughly studied, thus providing a natural standard for benchmarking pathway inference algorithms. We also chose to analyze two quantitative traits, fitness and reporter gene expression, to demonstrate that the method applies equally well to different phenotypes. While fitness is one of the most commonly analyzed traits [12]–[15], the use of gene expression, either in the form of microarrays [16]–[18] or fluorescent reporters [7], [19], is becoming more widespread [20].

We show that applying our method to fitness and expression traits provides complementary information. While the analysis of fitness provides information about the metabolic part of the network, the analysis of gene expression is required to infer how the network is regulated. When we combine the results of the two analyses, the method recovers nearly 80% of the known pair-wise relationships, without any false positives. This striking result suggests that our method can reliably extract important information about the causal relationships that define biological pathways and networks.

## Results

### Quantifying the effects of gene deletions

To quantify the phenotypic effects associated with single and double gene deletions, we first denote the two genes as *X* and *Y*. In a given experiment, we measure a quantitative trait *T* as a function of a signal *S* that we control. The signal can be present or absent, and *X* and *Y* may be present or deleted. These experimentally controlled conditions are specified by the Boolean variables *s*, *x* and *y*, respectively. Correspondingly, for each gene pair, there are eight different experimental conditions where the trait is measured.

We quantify the effects of gene deletions on the trait using multilinear regression [1], [11], [21]. Mathematically, the regression equation used to analyze the eight experiments is given by: 
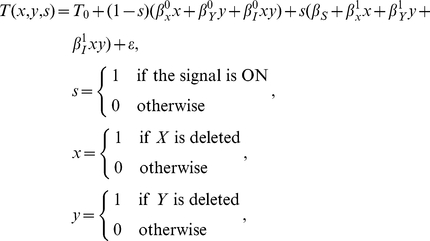
(1)where *ε* represents an error term. The regression parameters describe the trait value in the absence of the signal (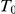), the effect of the signal in the absence of gene deletions (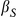), and, for each signal state, the impact of deleting gene *X* (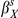) or gene *Y* (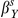), and an interaction term (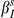). The interaction term captures the effect of deleting both genes that cannot be accounted by the effects of deleting the two genes individually.

### Identifying genetic interactions and epistasis

Equation (1) is consistent with a commonly used approach to identify genetic interactions when traits are quantified in a single environment or signal state. In this approach, a genetic interaction is inferred when the fitness trait *W* of the double mutant deviates from multiplicative neutrality, defined by *W_XY_W_wt_* = *W_X_W_Y_* [22]. Moreover, an epistatic genetic interaction is inferred when the trait of the double mutant is different from that of the wildtype (*W_XY_*≠*W_wt_*) and identical to one of the single mutants (*W_XY_* = *W_X_* or *W_XY_* = *W_Y_*).

Multiplicative neutrality is recovered from Equation (1) when the trait is defined as the log-transformed fitness. For example, in the ON signal state (*s = *1), the impacts of the signal and single gene deletions are given by:
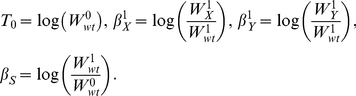
(2)


It follows from Equation (1) that the interaction term is given by: 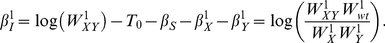
(3)


An equivalent neutrality function can be derived for the OFF signal state (*s = *0). In both cases, a genetic interaction is identified when *β_I_*≠0, and this interaction is epistatic when *β_I_* = −*β_X_* or when *β_I_* = −*β_Y_*.

### A quantitative model for interpreting epistasis

To develop a quantitative pathway inference method, we use a theoretical model to predict the effects of gene deletions within different signal-responsive hierarchical pathways (Table 1A). We consider eight pathway architectures, corresponding to activation or repression at each of three pathway steps. To predict the theoretical impact of gene deletions, we assume that gene *X* is upstream of gene *Y*. Later, we will use these predictions to interpret experimental data obtained without knowing the identity of the upstream gene or whether the genes even interact. In this case, we must discriminate among 16 possible pathways, as well as a null model corresponding to no interaction.

**Table 1:** Hypothetical pathways and predicted values of experimental parameters.

A	1***S*↓*X*↓*Y*↓*T***	2***S*↓*X*↓*Y*⊥*T***	3***S*↓*X*⊥*Y*↓*T***	4***S*↓*X*⊥*Y*⊥*T***	5***S*⊥*X*↓*Y*↓*T***	6***S*⊥*X*↓*Y*⊥*T***	7***S*⊥*X*⊥*Y*↓*T***	8***S*⊥*X*⊥*Y*⊥*T***
**B**	Predicted parameter values							
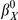	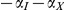		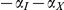		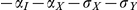		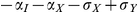	
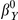	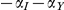		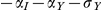		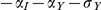		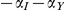	
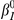	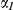		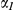		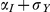		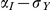	
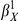	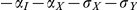		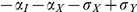		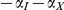		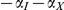	
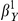	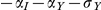		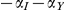		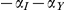		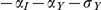	
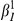	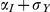		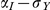		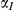		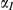	

(**A**) Hypothetical pathway architectures defining signal-dependent relationships.

(**B**) The definitions of *β*-parameters in terms of influence parameters predicted from the model in Figure 1.

The theoretical model, which is illustrated in Figure 1, uses the Boolean variables *x*, *y* and *s* to describe the experimental conditions, and two Boolean variables *x_S_* and *y_S_* to describe the respective signal-specific activities of gene *X* and gene *Y*. We assume that *x_S_* and *y_S_* behave in accordance with the relationships depicted in Table 1A. Correspondingly, in the absence of gene deletions, *x_S_* is determined exclusively by the signal *s*, and *y_S_* is determined exclusively by *x_S_*. In the presence of gene deletions, the signal-specific activities are defined by the following rules:

**Figure 1:**
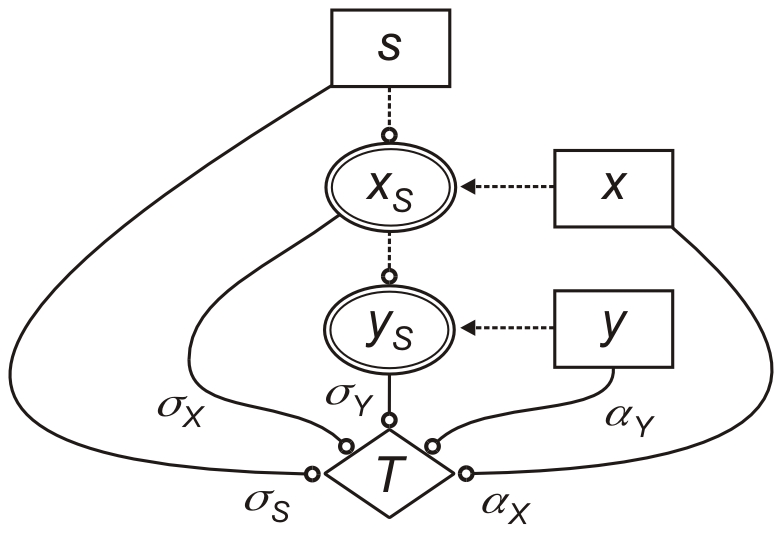
Theoretical model used to interpret epistasis in signal-responsive pathways. Lines ending in circles indicate influences that can be activating or repressing. Dotted lines indicate Boolean relationships defined in Eq. (4). Variables and influences are defined in the text (*α_I_* is omitted for clarity).



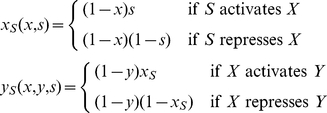
(4)The rules simply state that the signal-specific activity of each gene is present only when it is not deleted and not inactivated by the factor directly upstream. They also define the signal-dependent genetic interaction between the two genes.

The model uses three signal-specific influences, *σ_S_*, *σ_X_* and *σ_Y_*, and three signal-independent influences, *α_X_*, *α_Y_* and *α_I_*, to capture how the different model variables contribute to a theoretical trait (see Figure 1). While the signal-independent influences reflect the basal functions of the genes, the signal-specific influences *σ_S_*, *σ_X_*, and *σ_Y_* provide quantitative information about pathway function. A non-zero value of *σ_Y_* indicates that the pathway is involved in the cellular response to the signal, and correspondingly, pathways with higher values of *σ_Y_* contribute more to the response. A non-zero value of *σ_X_* implies a pathway branch point where the upstream gene affects the trait independently of the downstream gene. The absence of such a feedforward loop indicates that the two genes encode factors that function as a single entity (e.g., different subunits of a protein complex). Lastly, a non-zero value of *σ_S_* indicates that the effect of the signal is mediated through multiple pathways, and not defined exclusively by the two genes being analyzed.

The six influences can contribute to the theoretical trait value *θ* in a manner that depends on the environmental conditions and the pathway architecture as follows: 
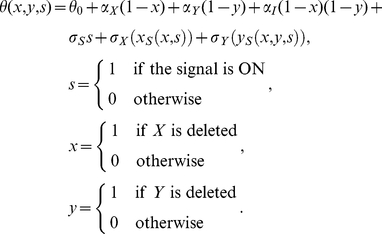
(5)


Here, *θ*_0_ is a baseline trait value, and *x_S_*(*x*,*s*) and *y_S_*(*x*,*y*,*s*) define the architecture of the pathway in accordance with the rules in Eq. (4). Correspondingly, the signal-specific influences *σ_S_*, *σ_X_*, and *σ_Y_* contribute to the trait only when the signal is present, and only when the two genes are active, respectively. In contrast, the signal-independent influences *α_X_* and *α_Y_* contribute to the value of the trait whenever *X* and *Y* are not deleted, respectively, and their basal interaction, *α_I_*, has an effect only when both genes are present.

We can now derive expected effects of gene deletions by equating the measured trait *T* defined in Eq. (1) and the theoretical trait *θ* defined in Eq. (5) for different pathway architectures and experimental conditions. The result is provided in Table 1B (see Table S1 for details), which gives the definitions of *β*-parameters in terms of the signal-specific and signal-independent influences. The predicted *β*-parameters are defined identically for pathways that differ only by the downstream gene having a positive (*σ_Y_*>0) or negative (*σ_Y_*<0) signal-specific effect on the trait. We also note that for each pair of *β*-values (i.e., the two values of each 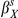, 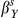 and 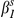), one of two values is always defined entirely by the basal, signal-independent effect of the gene deletions.

### Recovering the Avery-Wasserman inference rules

Before addressing the pathway inference problem, we examined the model to determine the assumptions required to recover the rules deduced by Avery and Wasserman. From Table 1B, it is immediately apparent that Rule (1) can be recovered when genes have no signal-independent functions (i.e., *α_X_ = α_Y_ = α_I_* = 0). Because one of each 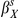, 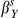 and 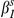 is defined exclusively by *α_X_*, *α_Y_*, and *α_I_*, gene deletions will in this case have an impact when the signal is either ON or when it is OFF, but not both.

To determine the applicability of Rule (2) and Rule (3), we examined the values of the *β*-parameters predicted when Rule (1) is valid by setting all signal-independent influences equal to zero. These values are given in Table 2A. In this case, gene deletions affect the trait in the same signal state when the upstream gene activates the downstream gene (i.e., a ‘positively regulated pathway’), and in opposite signal states when the upstream gene represses the downstream gene (i.e., a ‘negatively regulated pathway’). Thus, we recover the parts of Rule (2) and Rule (3) that govern the inference of causality between the two genes.

**Table 2:** Predicted values of experimental parameters used for inferring pathway architecture and gene order.

	Pathways 1 & 2	Pathways 3 & 4	Pathways 5 & 6	Pathways 7 & 8
**A**	Difference parameters			
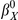	0	0	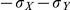	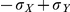
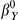	0	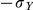	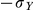	0
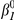	0	0	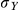	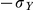
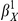	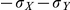	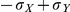	0	0
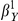	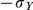	0	0	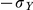
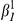	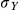	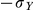	0	0
**B**	Difference parameters			
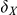	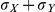	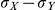	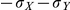	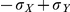
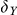	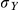	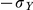	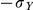	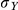
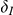	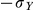	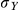	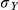	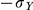

(**A**) Predicted *β*-parameters after the correction for signal-independent effects.

(**B**) Predicted *δ*-parameters obtained by analyzing difference traits.

Interestingly, by analyzing the model, we find that the parts of Rule (2) and Rule (3) governing which gene masks the other depend differentially on the presence of a feedforward loop. Within negatively regulated pathways, masking of the upstream gene can only be observed if the value of the influence *σ_X_* is zero, since this is the only case where *β_I_* = −*β_X_*≠0 (Pathways 3, 4, 7 and 8 in Table 2A). Therefore, Rule (2) is only applicable if the upstream gene acts exclusively through the downstream gene. Conversely, for positively regulated pathways, masking can only be observed if *σ_X_* is non-zero, since this is the only case where *β_I_* = −*β_Y_*≠0 (Pathways 1, 2, 5 and 6 in Table 2A). Therefore, Rule (3) is only applicable when the upstream gene influences the trait independently of the downstream gene.

### A general rule for inferring gene order

Since the Avery-Wasserman rules are not generally applicable, we re-examined the *β*-parameters predicted when no assumptions are made (Table 1B). We found that taking the difference between *β*-parameters in opposite signal states could eliminate all signal-independent influences. This correction for basal gene deletion effects yields three differential parameters, 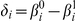 for *i* = (*X*, *Y*, *I*), that are defined exclusively by signal-specific influences (Table 2B). They quantify the signal-dependent effects of gene deletion (*δ_X_* and *δ_Y_*) and the signal-dependent effect of the genetic interaction (*δ_I_*).

Moreover, for all pathways, the model predicts that the effect of deleting the downstream gene (*δ_Y_*) is negated by the interaction term (*δ_Y_* = −*δ_I_*). We can therefore formulate a single, general rule for inferring gene order:

- *When the deletion of one gene masks the signal-dependent effect of deleting another*, *the masked gene is downstream irrespectively of the pathway architecture*.

Correspondingly, in the experimental analysis of two arbitrary genes, *A* and *B*, we can determine their order by evaluating if *δ_A_* = −*δ_I_* or if *δ_B_* = −*δ_I_* without making any assumptions about the data.

The *δ*-parameters capture the effects of gene deletions on the difference trait, *D*, defined as the change in trait values when the signal is absent and present, *D*(*x*,*y*) = *T*(*x*,*y*,0)−*T*(*x*,*y*,1). Accordingly, when *T* is the log-transformed fitness, *D* is the log-transformed sensitivity, *S*, since, by definition, log(*S*) = log(*W* ^0^)−log(*W* ^1^). Moreover, the relationship between the *δ*-parameters and *D* is given by the regression equation: 
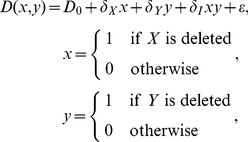
(6)


where *D_0_* is the base-line difference trait. In other words, *δ_X_* and *δ_Y_* capture the effect of single gene deletion on the ‘sensitivity’ or difference trait, while *δ_I_* captures the impact of the genetic interaction with respect to this trait.

The use of *δ*-parameters for the identification and interpretation of genetic interactions is not without precedent. St-Onge et al.[14] argued that examining the sensitivity of deletion mutants enables a focus on pathways responding to cellular changes, and used sensitivity to sub-classify genetic interactions involved in the response to drug treatment. Subsequently, Batenchuk et al. [23] demonstrated that multiplicative neutrality based on sensitivity phenotypes, *S_XY_S_wt_* = *S_X_S_Y_*, can be used to quantify how genetic interactions change in response to environmental perturbations. This analysis corresponds to determining the signal-dependent genetic interaction term *δ_I_*: 
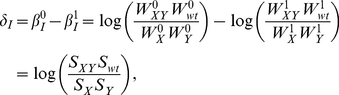
(7)


where superscript indicates whether the signal *s* is ON (*s* = 1), or OFF (*s* = 0). An equivalent approach was recently used by Bandyopadhyay et al. [24] to map how a genetic interaction network involving hundreds of genes is modulated by the presence of a drug.

### Inferring the architecture of signal-responsive pathways

It is straightforward to discriminate among the different hypothetical pathways when gene order has been determined, provided that genes have no signal-independent functions. In this case, we can narrow down the number of plausible pathways from eight to two by comparing the measured *β*-parameters obtained from experimental data using Equation (1) to those predicted when *α_X_*, *α_Y_* and *α_I_* are equal to zero (Table 2A). The most general approach to match experimental parameters to those predicted for a hypothetical pathway is to determine the signal states where one of the two experimental values of *β_X_*, *β_Y_* and *β_I_* is equal to zero. This is because the relationships involving the signal and the two genes can be identified uniquely by the signal states where the genes are inactive (see Table 2A). Correspondingly, the inference of pathway architecture does not require the use of statistical model discrimination methods. Lastly, whether the downstream gene increases (*σ_Y_*>0) or decreases (*σ_Y_*<0) the trait can be inferred directly from the sign of the experimental non-zero interaction term. For example, for pathways 3 and 4, the influence is predicted to have the opposite sign of the measured interaction term (i.e. *β_I_* = −*σ_Y_*), and therefore pathway 3 would be inferred if the measured interaction was negative or pathway 4 if it was positive (see Table 2A).

The pathway inference method described above can also be applied when genes have signal-independent functions. In this case, however, the definitions of the *β*-parameters in Table 2A correspond to corrected *β*-values, obtained by subtracting the basal effects of deletions associated with each parameter. As noted earlier, one of two measured values of *β_X_*, *β_Y_* and *β_I_* always corresponds to this basal effect (see Table 1B). The problem is to identify whether the basal effect is observed when the signal is OFF or ON.

To obtain corrected *β*-parameters, we assume that the basal effect of gene deletion corresponds to the experimental *β*-value with the lowest magnitude. This assumption is valid for genes whose primary biological function is associated with a signal-responsive pathway. The assumption is also valid when the activating or repressing functions of the gene are the same in the presence and absence of the signal. In this case, the effect of losing both basal and signal-specific gene functions is necessarily greater in magnitude than the loss of basal function alone (see Table S2).

While these two conditions should cover most instances, there are cases where the assumption may introduce false inferences, for example when a gene functions as a repressor when the signal is OFF and as an activator when the signal is ON. One of the central genes in the galactose pathway actually displays such dual functionality with respect to fitness (see below). However, a false inference is not made since the pattern of corrected *β*-values does not match any of those listed in Table 2A.

### Quantifying causal relationships from measured trait values

Once the pathway has been inferred, the signal-independent and signal-specific influences in Figure 1 can be calculated directly from measured trait values using the definitions given in Table 3. In most cases, the definitions of the influences are intuitive. For example, in pathways where the upstream gene is an activator, the signal-independent influence of the upstream gene (*α_X_*) is given by the effect of deleting gene *X* when gene *Y* and the signal are absent. Similarly, the signal-specific influences *σ_Y_* and *σ_X_* can be calculated using the difference traits. They are defined by the change in *D* caused by deleting *Y* relative to wildtype, and the change in *D* caused by deleting *X* when *Y* is absent, respectively.

**Table 3:** Definitions of influences for hypothetical pathways based on the values of the trait *T* and the difference trait *D*.

	Pathways 1 & 2	Pathways 3 & 4	Pathways 5 & 6	Pathways 7 & 8
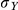	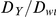	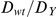	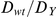	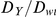
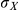	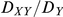	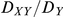	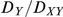	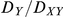
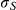	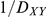	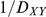	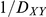	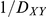
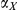	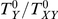	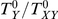	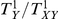	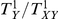
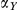	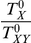	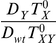	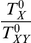	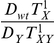
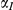	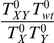	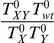	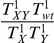	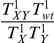

The trait values are not log transformed for clarity.

### A step-wise inference method

The three previous sections have addressed the inference of gene order, pathway architecture and quantitative influences. We used the results from these analyses to develop an inference algorithm that can be applied to experimental data. The following steps summarize the algorithm as applied to two arbitrary genes *A* and *B*:

**Step 1:** Determine if the two genes have a signal-dependent interaction, i.e., if *δ_I_*≠0.

**Step 2:** Evaluate if *δ_A_* = −*δ_I_≠δ_B_* or if *δ_B_* = −*δ_I_*≠*δ_A_* to determine whether *A* or *B* is the downstream gene.

**Step 3:** Correct the measured *β*-parameters for basal, signal-independent effects and infer the pathway by matching the corrected values to those given in Table 2A.

**Step 4:** Calculate the influence parameters using Table 3.

We note that Step 2 restricts the analysis to pathways where the upstream gene influences the trait independently of the downstream gene and *σ_X_*≠0 (see Table 2B). When the effect is mediated exclusively through the downstream gene (*σ_X_* = 0), the single deletion mutants have the same trait values and there is no unique masking interaction (*δ_B_* = *δ_A_* = −*δ_I_*). Order can in this case be identified in Step 3 if the non-zero impact parameters (*β_A_* and *β_B_*) are found in opposite environments (corresponding to a negatively regulated pathway, see Table 2A). If this is not the case, the two genes are inferred to act as a cohesive unit. We also note that there are several ways to evaluate equivalence in Step 2. In our experimental benchmarking of the method (see below), we determine epistasis by evaluating if the difference between the observed (i.e., *δ_I_*) and the predicted interaction term (i.e., −*δ_A_* or −*δ_B_*) is smaller than the experimental error (see Materials and Methods). However, other methods should apply equally well.

### Experimental benchmarking using the *GAL* network

**Figure 2:**
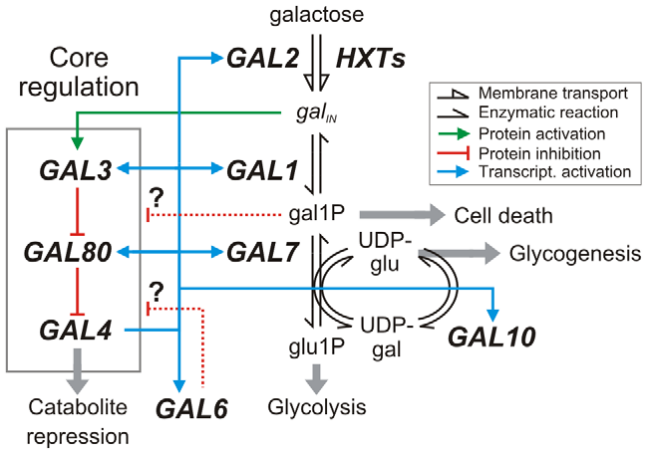
Canonical yeast galactose utilization pathway (adapted from[26]). Gray arrows indicate cellular processes likely impacted by *GAL* gene deletions. Abbreviations: intracellular galactose (gal*_IN_*), galactose-1-phosphate (gal1P), glucose-1-phosphate (glu1P), uridine diphosphate (UDP), UDP-glucose (UDPglu), UDP-galactose (UDPgal).

To benchmark the method, and to critically assess fitness- and expression-based epistasis analyses in general, we investigated a thoroughly characterized network, the yeast galactose utilization pathway. This network, which is depicted in Figure 2, involves three regulatory genes (*GAL3*, *GAL4* and *GAL80*) and five structural genes (*GAL1*, *GAL2*, *GAL6*, *GAL7* and *GAL10*) that enable yeast to detect and metabolize galactose. Quantitative traits were measured for a library of single and double *GAL* gene deletion strains in a genetic background expressing yeast enhanced green fluorescent protein (*yEGFP*) from the promoter of the *GAL10* gene (see Materials and Methods). Growth rates during early log-phase (fitness) and reporter expression were determined in rich media containing raffinose under inducing (+galactose; “ON”) and non-inducing (-galactose; “OFF”) conditions (see Materials and Methods).

The *GAL* network consists of a core regulatory branch and a metabolic branch. The protein encoded by *GAL4* (Gal4p) is considered the main regulator of the network. It binds to the regulatory regions of all other *GAL* network genes, but remains inactive in the absence of galactose due to repression by Gal80p. Intracellular galactose induces the expression of the *GAL* structural genes by activating Gal3p, which subsequently relieves the repression of Gal4p by Gal80p. Since Gal4p binds to the promoters of *GAL3* and *GAL80*, the network contains both a positive and a negative feedback loop.

The structural *GAL* genes are involved in converting intracellular galactose into glucose-1-phosphate (glu1P) in a process that involves galactose-1-phosphate (gal1P), UDPglu and UDPgal (see Figure 2 for details). Some of the structural genes and metabolic intermediates have been implicated in the regulation of the network. The *GAL2* gene encodes a trans-membrane transporter, which, together with transporters encoded by the *HXT* genes, allows galactose to enter the cell and activate Gal3p. This establishes a second positive feedback loop since Gal4p binds to the promoter of *GAL2*. The deletion of *GAL6* and *GAL7* has been reported to change the expression of other *GAL* genes [25], [26], suggesting that Gal6p and gal1P may have regulatory functions. However, these roles are not firmly established.

### Most *GAL* genes have galactose-independent functions

**Figure 3:**
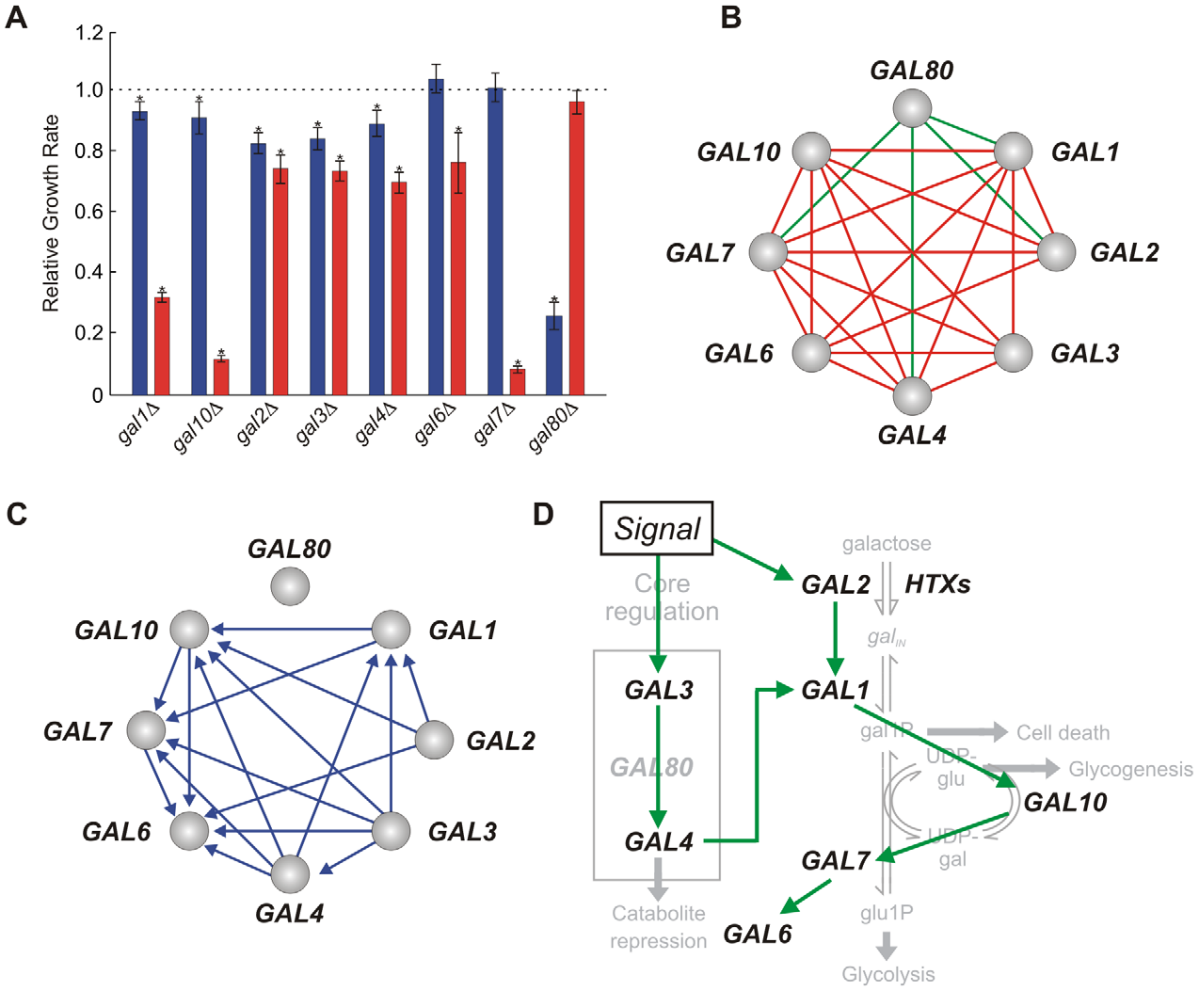
Analysis of fitness traits. (**A**) Relative fitness for single *GAL* gene deletions, in presence (red) and absence (blue) of galactose. Asterisk marks significant effect (t-test *p*-value <0.05). (**B**) Signal-dependent genetic interactions (green: *δ_I_*>0, red: *δ_I_*<0, t-test *p*-value <0.05). (**C**) Inferred causal relationships. (**D**) Transitive reduction of the network in (C).

As expected, the deletions of individual *GAL* genes (*GAL80* excepted) are associated with significant reductions in fitness in the presence of galactose (Figure 3A). While the deletions of *GAL1*, *GAL10* or *GAL7* resulted in severe sickness and fitness reductions of 68%, 88% and 92%, respectively, the deletions of *GAL3* or *GAL4* resulted in relatively mild defects and fitness reductions of ∼30%. The severity of the *GAL7* deletion can be attributed to a combination of pathway disruption and accumulation of the toxic intermediate gal1P [27]. Accumulation of gal1P may also explain the severity of the *GAL10* deletion since Gal10p is required to replenish the UDPglu consumed in the conversion of gal1P into glu1P.

Unsurprisingly, most of the single deletions are also associated with significant fitness defects under the non-inducing condition (Figure 3A), and their interactions cannot be analyzed using the Avery-Wasserman rules. These defects, which range between reductions of 7% in the *gal1*Δ mutant to 74% in the *gal80*Δ mutant, indicate that the *GAL* genes have important activities even in the absence of galactose. The severity of the *GAL80* deletion is not intuitive since expression of the structural *GAL* genes should not be this detrimental when galactose is absent. The effect is due to the activation of Gal4p, since deleting *GAL4* in the *gal80*Δ mutant results in the restoration of normal growth in the absence of galactose (see below). A plausible explanation is that Gal4p effectively shuts down the utilization of the alternate carbon source, raffinose. This phenomenon, called catabolite repression [28], also explains the severity of the *GAL1* deletion in the presence of galactose. In this case, Gal4p is fully active, but neither galactose nor the alternate carbon source can be metabolized.

### Fitness traits reveal metabolic pathways and relationships

We analyzed fitness traits by applying the step-wise inference method described above (Figure 3). We found that 23 of the 28 gene pairs had a galactose-dependent genetic interaction (Figure 3B, Table S3). Gene order could be determined for 18 out of 23 pairs by identifying signal-dependent epistasis based on *δ*-parameters. In 17 of these cases, the patterns of zero-valued experimental *β*-parameters, once corrected for basal effects, matched the pattern predicted for Pathway #1 in Table 1A. The regulatory relationships inferred for the 17 gene pairs are shown in Figure 3C.

The only epistatic interaction that is inconsistent with a hypothetical pathway is the one involving *GAL4* and *GAL80*. This is likely due to Gal4p functioning as both an activator and a repressor of fitness. While Gal4p contributes positively to growth in the presence of galactose, activating Gal4p by deleting *GAL80* causes a severe fitness defect in the absence of galactose.

### Pair-wise causal relationships contribute to a global network model

The establishment of network models from genetic interaction data is a complex problem (see Battle et al. [7]). Here, we focus on the problem of extracting a directed graph incorporating casual upstream/downstream relationships inferred between gene pairs that exhibit epistasis. Correspondingly, the graph only contains genetic interactions that conform to our model.

The most straightforward approach is to generate the transitive reduction of the network diagram containing all inferred linkages [29]. This approach eliminates the shorter of two paths connecting any two genes. For example, suppose that in the analysis of three genes, *A*, *B* and *C*, *A* is inferred upstream of *B* and *C*, and *B* is inferred upstream of *C*. Here, the transitive reduction will imply that the influence of *A* on *C* is indirect through *B*. This approach yields the simplest network diagram consistent with all upstream/downstream relationships. Because our inference of pathway architecture is contingent on each gene being regulated by a single upstream factor, the use of transitive reduction is justified.

The global network model generated by transitive reduction is shown in Figure 3D. Here, we infer the correct order and dependency among the structural genes *GAL1*, *GAL2*, *GAL7* and *GAL10*. The deletion of *GAL2* masks the effects of deleting *GAL1* or *GAL10*, correctly identifying Gal2p as the first enzyme in the metabolic cascade. Similarly, the deletion of *GAL1* masks the impact of deleting *GAL10* or *GAL7*, and Gal1p is correctly identified as the second enzyme in pathway. Lastly, deleting *GAL10* masks the impact of deleting *GAL7*, placing Gal10p upstream of Gal7p.

We also correctly infer Gal4p as an activator of all the structural genes. The placement of *GAL4* upstream of *GAL1* is consistent with complete disruption of galactose metabolism when *GAL4* is deleted. However, it is inconsistent with protein-DNA and transcriptional data demonstrating that Gal4p directly activates the transcription of all the structural genes independently of *GAL1*. Indeed, the analysis of epistasis reveals functional rather than physical relationships. For example, the inference of *GAL4* upstream of *GAL7* is not due to the fact that Gal4p is required for *GAL7* expression. If this were the case, the *gal7*Δ and the *gal4*Δ*gal7* Δ mutants would have similar phenotypes. Instead, deleting *GAL4* rescues the severe fitness defect of deleting *GAL7* by preventing *GAL1* expression and, therefore, gal1P accumulation.

### Pathway influences imply multiple sources of galactose toxicity

**Figure 4:**
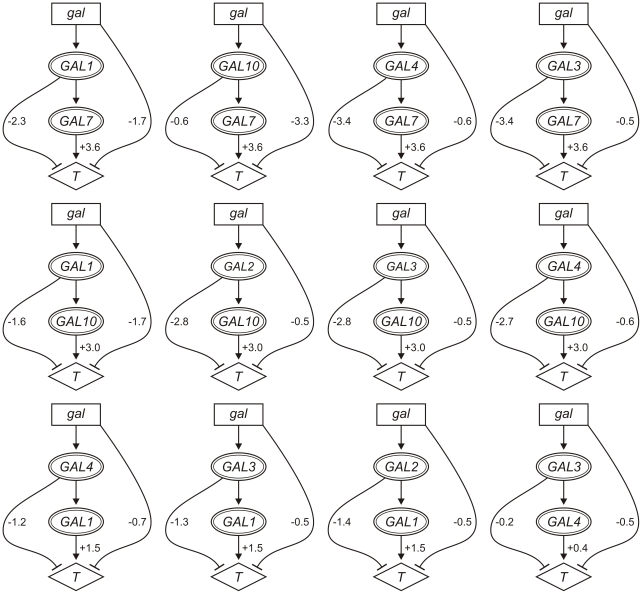
Quantitative pathway inference using fitness traits. Relationships inferred between the signal (*gal*) and *GAL* genes are shown with values of their signal-specific influences (95% confidence) on fitness (*T*). Pathways involving *GAL6* are omitted.

Once gene order and pathway architecture was established between two genes, we calculated the influences involved in each pathway using the definitions in Table 3. The resulting pathway models are shown in Figure 4, where the values of the pathway-dependent (*σ_Y_*), feedforward (*σ_X_*) and pathway-independent (*σ_S_*) influences allow for a quantitative interpretation of pathway function. A complete list of the calculated influence parameters and 95% confidence intervals is in Table S4.

Strikingly, the quantities of the influences parameters demonstrate that the effect of galactose is not mediated through a single gene or pathway step. Most inferred pathways have significant repressing feedforward and pathway-independent influences (95% confidence), resulting in incoherent feedforward architectures. In the context of metabolic pathways, such influences quantify the effect of accumulation of a toxic metabolic intermediate produced by the upstream enzyme, or by the loss of a beneficial intermediate. For example, the effect of galactose accumulation [30] is captured by the pathway-independent influence of the signal in pathways where *GAL4* is the upstream gene (*σ_S_* = −0.6). Accumulation of the second metabolite in the pathway, gal1P, can be attributed to the feedforward influence in the *GAL1*/*GAL7* pathway (*σ_X_* = −2.3). This accumulation is less profound when captured by the feedforward influence in the *GAL1*/*GAL10* pathway (*σ_X_* = −1.7), presumably because alternative sources of UDPglu are available to convert gal1P to glu1P in the absence of Gal10p ([31], [32], see Figure 2). The effect of imbalance in UDPglu pools can be attributed to the feedforward influence in the *GAL10*/*GAL7* pathway (*σ_X_* = −0.6). Moreover, the effect of catabolite repression is captured by the feedforward influence in the *GAL4*/*GAL1* pathway (*σ_X_* = −1.2). Interestingly, fitness is more severely affected by perturbation of toxic galactose-derived metabolites (e.g., *σ_Y_* = 3.6 in pathways where *GAL7* is the downstream gene) than complete disruption of galactose metabolism (i.e.,*σ_Y_* = 0.4 in the *GAL3*/*GAL4* pathway).

### Expression traits identify transcriptional regulators of the *GAL* network

**Figure 5:**
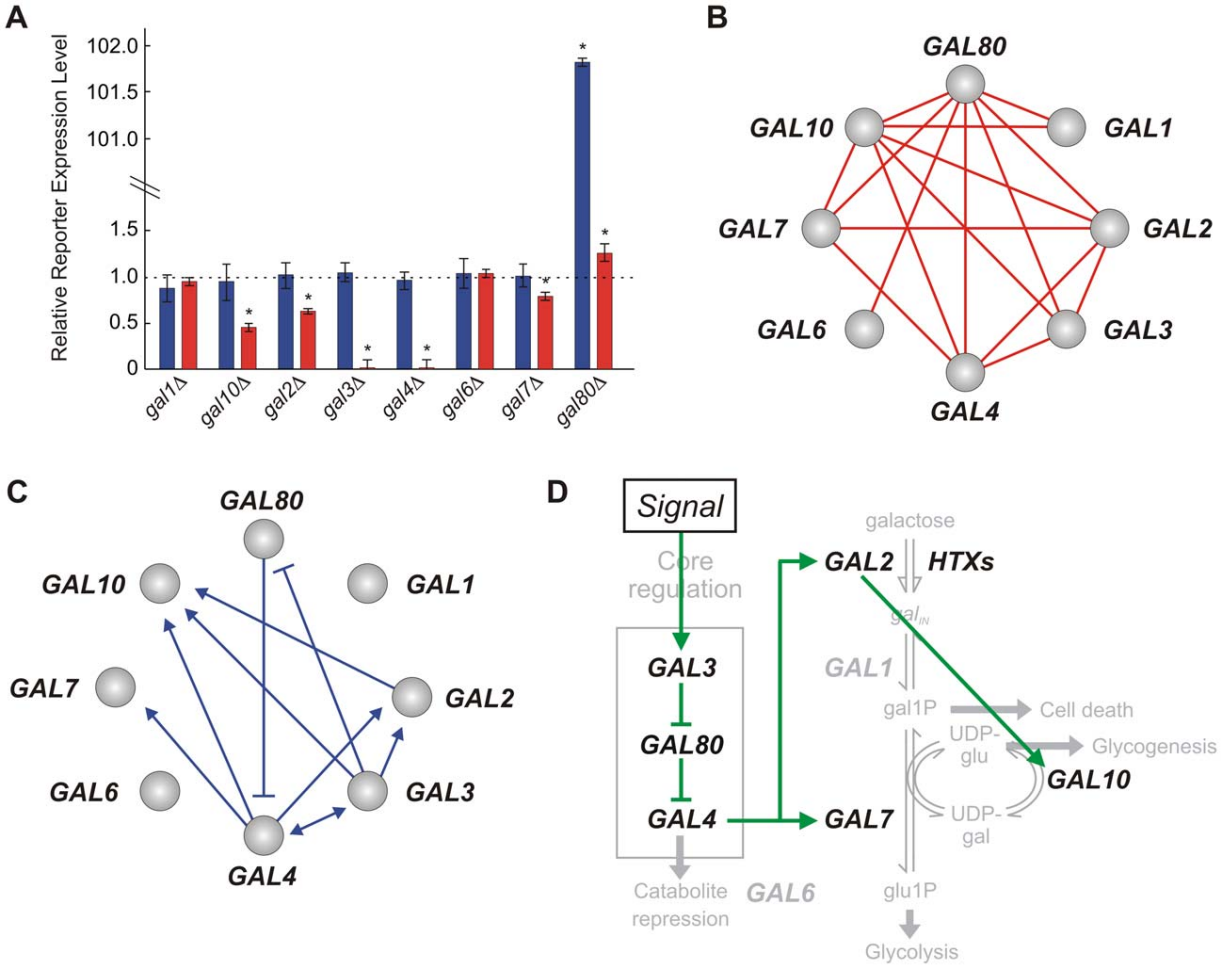
Analysis of reporter expression traits. (**A**) Relative traits for single *GAL* gene deletions, in presence (red) and absence (blue) of galactose. Asterisk marks significant effect (t-test *p*-value <0.05). (**B**) Signal-dependent genetic interactions (green: *δ_I_*>0, red: *δ_I_*<0, t-test *p*-value <0.05). (**C**) Inferred causal relationships. (**D**) Transitive reduction of the network in (C).

We applied the step-wise inference method to reporter expression traits exactly as described for fitness phenotypes (Figure 5). Single deletion expression traits, measured in the presence and absence of galactose, are shown in Figure 5A. The traits of the *gal1Δ* and *gal6Δ* mutants are indistinguishable from that of the wildtype strain, suggesting that these genes do not contribute to transcriptional regulation of the reporter. Most of the other *GAL* genes have a statistically significant impact. Notably, the deletion of *GAL3* or *GAL4* causes complete pathway deactivation (decrease by 98%) in the presence of galactose. This identifies *GAL3* and *GAL4* as central network activators. Similarly, the central regulatory function of *GAL80* is reflected by the phenotype of the *gal80Δ* mutant, which displays a 100-fold increase in expression in the absence of galactose.

Surprisingly, the deletions of many structural genes (*GAL2*, *GAL7* or *GAL10*) also cause a statistically significant decrease of reporter expression under inducing conditions. The decrease in the *gal7Δ* and *gal10Δ* strains could arise from the severe growth defects of these mutants [33]. We ruled out this possibility by examining reporter expression driven by the constitutive *P*_ACT1_ promoter. For both the *gal7Δ* and *gal10Δ* mutants, *P*_ACT1_ reporter expression was increased rather than decreased compared to the wildtype (data not shown). This suggests that *GAL7* and *GAL10* are involved in modulating regulatory signals within the network.

### Expression traits reveal regulatory pathways and interactions

By applying the step-wise inference method to reporter expression traits, we identified 17 signal-dependent genetic interactions (Figure 5B, Table S3), of which 9 were epistatic and consistent with a hypothetical pathway. The resulting regulatory relationships inferred are shown by means of an acyclic graph in Figure 5C.

Performing transitive reduction of the graph in Figure 5C allows for full recovery of the core regulatory branch of the network, in the form of a linear cascade containing *GAL3*, *GAL80* and *GAL4* (Figure 5D). Notably, the expression phenotypes of the *gal3Δ* and *gal4Δ* mutants are indistinguishable, and hence the order between *GAL3* and *GAL4* cannot be inferred based on this relationship alone. However, it can be deduced from the inferred relationships between *GAL3* and *GAL4* with *GAL80*. While *GAL3* is correctly inferred to repress *GAL80*, *GAL80* is correctly inferred as a repressor of *GAL4*. Correspondingly, *GAL3* must be upstream of *GAL4,* and act on *GAL4* indirectly through *GAL80*. The analysis also correctly identifies *GAL4* as an activator of *GAL2*, *GAL7* and *GAL10*, implicating both *GAL3* and *GAL80* as regulators of these genes (Figure 5D).

The analysis also identifies epistatic relationships among the structural *GAL* genes. Specifically, *GAL2* is identified as an upstream activator of *GAL10*, and *GAL1* is identified upstream of *GAL10.* The pathway involving *GAL1* and *GAL10* cannot be inferred because the deletion of *GAL1* has no differential impact (*δ_X_* = 0). However, order can be inferred since deleting *GAL1* completely masks the effect of deleting *GAL10*. This implies that a metabolic intermediate downstream of *GAL1* and upstream of *GAL10* has a regulatory role in the *GAL* pathway. With this in mind, the interaction between *GAL2* and *GAL10* may be explained by a reduced rate of galactose influx, preventing the concentration of this regulatory metabolite from being significantly perturbed.

### Pathway influences on expression distinguish major and minor regulatory pathways

**Figure 6:**
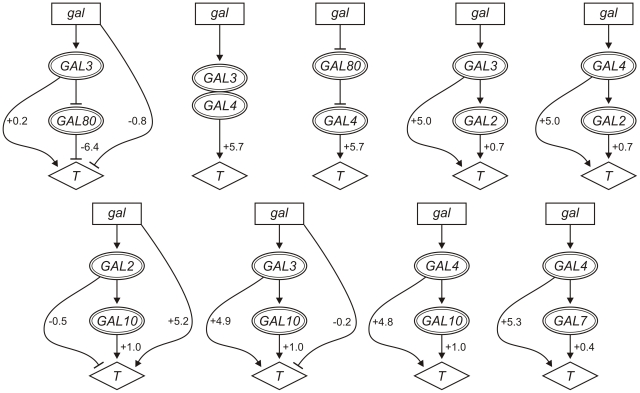
Quantitative pathway inference using expression traits. Relationships inferred between the signal (*gal*) and *GAL* genes are shown with values of their signal-specific influences (95% confidence) on expression (*T*).

In Figure 6, we depict the significant contributions of the signal-specific influences in pathways inferred based on the expression trait. A complete list of mean influence parameters and 95% confidence intervals is given in Table S5.

In contrast to the analysis of fitness traits, we infer that the effect of galactose on reporter expression is mediated almost exclusively through the linear cascade involving *GAL3*, *GAL4*, and *GAL80*. The pathway-independent influence *σ_S_* is negligible or small in pathways where *GAL3*, *GAL4* or *GAL80* is the upstream gene (Figure 6). In addition, *GAL3*, *GAL4* and *GAL80* are identified as forming a cohesive regulatory unit since the feedforward influence *σ_X_* is negligible in all pathways involving these genes. The pathways involving *GAL2* and *GAL10* provide the contrast to these observations. The *GAL2/GAL10* pathway mediates only a minor effect, and the feedforward influence is consistently high for pathways where *GAL2* or *GAL10* is the downstream gene.

Surprisingly, the regulatory effect mediated through *GAL7* (*σ_Y_* = 0.4) is less than that mediated through *GAL10* (*σ_Y_* = 1). The hypothesis that gal1P has regulatory functions would predict the opposite result since gal1P should accumulate to higher levels in the *gal7Δ* mutant than in the *gal10Δ* mutant (see Figure 2). Correspondingly, it seems likely that the perturbed regulatory metabolite is UDPglu or UDPgal rather than gal1P.

### Most known network interactions are recovered without false positives

A critical step in our method is the identification of signal-dependent epistasis (Step 2) where the signal-dependent effect of gene deletion is negated by the signal-dependent genetic interaction. In the above analysis, we identify signal-dependent epistasis by evaluating if the difference between the measured (*δ_I_*) and predicted (−*δ_X_* or −*δ_Y_*) values of the interaction term is smaller than a threshold value *ε_thr_* that we extract from the experimental data. Specifically, the threshold is defined by the relative standard error averaged over all significant interactions terms. The values of *ε_thr_* used in the analyses of fitness and expression traits were 13% and 20%, respectively.

To formally address the accuracy of our method, we systematically tested how varying the threshold used to identify epistasis impacts our inference. Notably, this is the only step in the algorithm that has a significant effect on the false positive rate. True epistatic interactions were predicted from the known *GAL* network topology (Figure 2), and consist of all well-established direct and indirect interactions between the *GAL* genes, including the four feedback loops (Gal4p activates Gal2p, Gal3p and Gal80p, and Gal7p is upstream of Gal10p).

**Figure 7:**
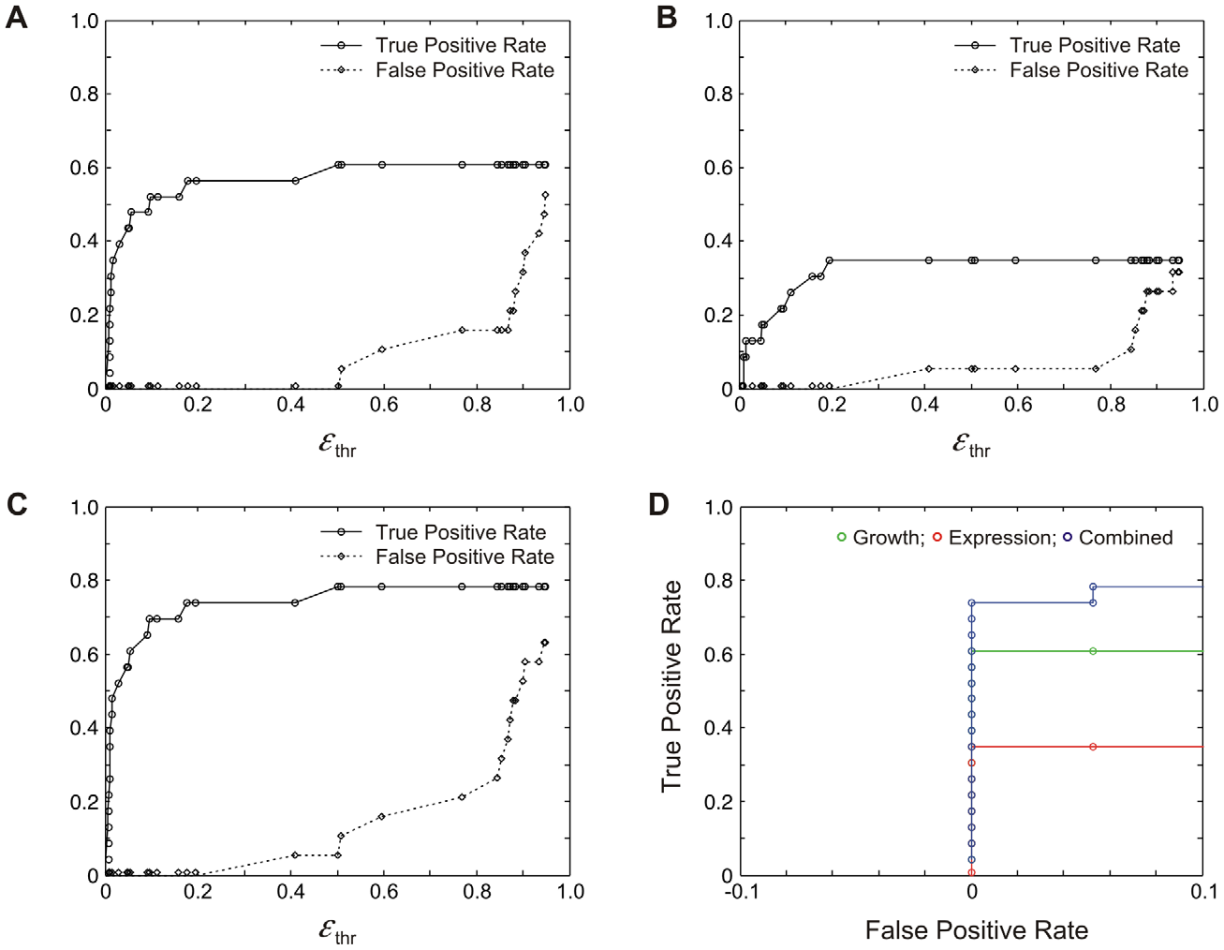
Analyses of true (TPR) and false positive rates (FPR) for inference of causal relationships among *GAL* genes. (**A, B**) TPR (circles) and FPR (diamonds) for analysis of fitness and expression, respectively. (**C**) TPR (circles) and FPR (diamonds) for combined analyses. (**D**) Receiver operating characteristic curves for fitness, expression and combined analyses.

The first two panels in Figure 7 demonstrate the effect on the true and false positive rates of changing the epistasis threshold in the analysis of fitness and expression traits, respectively. In both cases, the false positive rate is zero for a broad range of threshold values. False positives are only observed when the threshold reaches ∼50% for the fitness-based analysis (Figure 7A) and ∼40% for the expression-based analysis (Figure 7B). In both cases, the number of true positives plateau when the threshold is ∼20%. At this value of the threshold, the true positive rates for the analysis of fitness and expression traits is ∼60% and ∼30%, respectively. The reason a true positive rate of 100% is never reached is that additional criteria are imposed. Specifically, epistatic interactions are only inferred if a signal-dependent genetic interaction is observed, and if the involved genes have statistically significant effects when deleted individually (see Materials and Methods). Additionally, as expected, none of the known feedback loops are inferred.

To demonstrate the benefit of combining fitness- and expression-based epistasis analyses, we show in Figure 7C the true and false positive rates obtained by merging the results from the two datasets. Additionally, in Figure 7D, we compare the receiver operating characteristic (ROC) curves obtained from the individual analyses, and when they are combined. In both cases, it is apparent that considering both traits significantly improves the number of true positive relationships inferred while keeping the false positive rate low. Notably, when the threshold is kept below 20%, which is the case when it is extracted from the experimental error, nearly 80% of the interactions are correctly inferred with no false positives.

## Discussion

We have developed a quantitative model to facilitate the inference of causal relationships among genes functioning within signal-responsive pathways. The model generalizes the framework by Avery and Wasserman [8] where genes are strictly ON or OFF, with no intermediate levels of activity. Our model allows genes to have both signal-specific and signal-independent functions. It also allows the signal to influence the trait independently of the two genes, and the upstream gene to influence the trait independently of the downstream gene.

We used the model to develop a method to infer signal-responsive pathways from data generated in systematic gene deletion experiments. In this method, we first identify signal-dependent genetic interactions. This step is equivalent to recently developed methods referred to as ‘sensitivity-based epistatic analysis’ [23] or ‘differential epistasis mapping’ [24]. While the former identifies signal-dependent interactions based on the difference in mutant phenotypes in the presence and absence of the signal, the latter identifies these interactions by examining the change in genetic interaction strength caused by the signal. Next, we identify interactions where deleting one gene masks the signal-dependent effect of deleting the other. For these interactions, we can infer gene order since the masked gene is always the downstream gene when analyzing signal-dependent effects. Once gene order has been established, we determine the causal relationships within the pathway by matching the observed effects of gene deletions to those predicted for different hypothetical pathways.

When applied to experimental data obtained for the yeast galactose utilization pathway, the method recovers close to 80% of known causal relationships, with no false positives. The method can also be used to extract novel quantitative information about pathway function not made available by commonly used approaches that identify undirected functional relationships from fitness [12], [15], [24], [34] or expression data [35], [36]. For example, it automatically quantifies the relative effect of the signal mediated through parallel pathways, and the effect of the upstream gene independently of the downstream gene. This information in turn identifies pathway branch-points and provides weights to different pathway steps.

Our method is not without limitations. Notably, it is only applicable to data obtained using loss-of-function mutations, and, therefore, cannot reveal the feedback loops that are critical for a complete understanding of biological network function. Additionally, it can only infer pathways wherein gene activity is regulated by a single upstream factor. Further development is required to determine how the method may be applied to other types of pathways, including those containing functional and regulatory redundancies, and other types of genetic perturbations, such as partial loss-of-function, copy-number reduction and over-expression.

Network inference algorithms should in principle yield a concise model that accounts for all experimentally observed linkages while retaining only those corresponding to direct effects [36]. We resolved the issue of indirect effects by computing the transitive reduction [29] of the network containing all pairwise relationships. This is a valid approach since we apply it only to epistatic interactions that fully conform to our model. This restriction is likely why we make no false positive inferences and have no conflicts among the inferred interactions.

We anticipate that complex networks will contain more non-epistatic than epistatic interactions. Battle et al. [7] recently addressed this inference problem. In their approach, five types of pairwise hypothetical relationships, representing both epistatic and non-epistatic interactions, were used to generate putative network models. Each gene pair was assigned a consistency score based on the deviation of the observed double deletion phenotype from that predicted by the hypothetical relationship between the two genes. An optimization step was then applied to find an optimal network model by minimizing a global network score, aggregating the individual consistency scores within the network. We anticipate that our method can readily be integrated into such an approach and used to identify signal-dependent cascades and pathways within the global network architecture prior to optimization. Correspondingly, we anticipate that our method will play a key role in the establishment of quantitative network models from measurements of genetic interactions.

## Materials and Methods

### Strains and plasmids

To conduct an epistasis analysis of the *GAL* network, a library of 36 strains harboring *P_GAL10_*-*GFP* or *P_ACT1_*-*GFP* at the *ade2* locus and single or combinatorial deletions for all eight *GAL* genes was generated in haploid yeast *S*. *cerevisiae* (BY4742 MAT*α his3Δ1 leu2Δ0 lys2Δ0 ura3Δ0*, Open Biosystems). Briefly, the 1 kb region upstream of *GAL10* or *ACT1* was PCR amplified, digested and integrated upstream of *GFP* in a plasmid carrying *HIS3* and an ampicillin-resistance gene. Following integration into the *ADE2* locus using a PCR-based gene replacement strategy [37], reporter strains were selected by growth on yeast synthetic drop-out media (SC) without histidine containing 6.7 g/L yeast nitrogen base without amino acids (Wisent, Inc.), 1.92 g/L yeast synthetic drop-out media supplement without histidine (Sigma), and 20 g/L agar (Wisent, Inc.). Correct integration of each reporter construct was confirmed by PCR. Thereafter, single gene deletion strains were generated by replacing the corresponding loci with a Kanamycin resistance gene (*KanMX6*, [38]). Single mutants were selected by growth on SC without histidine and 0.3 g/L Geniticin (G418, Wisent, Inc.), and confirmed by PCR. Double gene deletion strains were generated from single deletion reporter strains, by replacing the second loci with *URA3*. Double mutants were selected by growth on SC agar plates without histidine and uracil, and confirmed by PCR. Yeast strains were stored at −80°C in yeast peptone dextrose (YPD) containing 10 g/L yeast extract and 20 g/L bacteriological peptone (Wisent, Inc.), supplemented with 2% (w/v) glucose (Sigma), 0.042 g/L adenine (Sigma), and 15% (w/v) glycerol (Sigma).

### Culture conditions for growth and reporter expression measurements

The library of 38 strains (including wildtype and a control strain lacking a reporter and deletions) were grown for two days at 30°C under continuous shaking (250 rpm) in Yeast Peptone Raffinose (YPR) media [26], and then stored for a maximum of four days in liquid culture at 4°C for experimental purposes. In each replicate experiment, 60 µl of 4°C stock cultures were used to inoculate a 96-well deep well plate containing 400 µl fresh YPR. Inoculated cultures were grown overnight (∼18 h) at 30°C under continuous shaking (250 rpm). Following overnight growth, the turbidity of each culture was measured using a Victor^3^V plate reader (PerkinElmer), and optical density (OD_600nm_) was adjusted to ∼0.15 by adding the appropriate volume of overnight culture into 300 µl of inducing (YPR with 2% w/v galactose) or non-inducing (YPR) media [26]. After 3 hours of growth, the optical density (OD) of cultures was readjusted by dilution to an OD of ∼0.02 in inducing or non-inducing media, in a final volume of 750 µl. Aliquots of 300 µl were taken from each media condition and plated in a 100-well honeycomb microplate (Growth Curves USA) wherein optical density was monitored over 22 h at 30°C using a Bioscreen C Analyzer (Growth Curves USA). The remaining 450 µl-cultures were kept at 30°C (250 rpm shaking) for 3 h, prior to expression analysis by flow cytometry. Four replicate experiments were conducted over a period of four days, for which growth rate and expression data were acquired to generate replicate data (four replicates for all combinatorial deletion strains, and eight replicates for all single deletion strains, wildtype and control).

### Quantification of growth rates

Optical density time courses were performed using a Bioscreen C Analyzer (Growth Curves USA). Turbidity in a 100-well honeycomb microplate was measured using a wideband filter (450–580 nm) every 15 min for 22 h at 30°C, without shaking. Growth rates were estimated by fitting OD values over time to an exponential growth model using MATLAB. Fits were restricted to OD values obtained within a window where reads are most accurate (0.1>OD<0.4) and a time interval corresponding to the timing of expression measurements conducted in parallel.

### Quantification of reporter expression

*P_GAL10_*-*GFP* reporter gene expression was quantified in individual cells using a Beckman-Coulter FC500 flow cytometer. A total of 60,000 events were collected for each sample and filtered using a custom software script in MATLAB where a fixed elliptical forward/side-scatter autogate was used to capture approximately 50% of the events. The fluorescence intensity (488 nm excitation, 510–550 nm emission) associated with these events was used to generate representative expression distributions for each sample. The means of these distributions were used as the reporter expression trait.

### Data analysis

Data analysis and pathway inference was conducted in MATLAB. A fully annotated inference script and the full dataset are available upon request. Replicate measurements of mean reporter expression and growth rates were log2 transformed and used to calculate experimental parameters quantifying the impacts of single gene deletions and interactions, by multilinear regression, as described in the results section. All significant single-deletion impacts and signal-dependent genetic interactions were identified using a *p*-value threshold of 0.05 (see Table S3).

To identify masking interactions, we calculated the absolute relative difference between the measured values of *δ_X_* or *δ_Y_* and *δ_I_*, using *ε_i_* = |*δ_i_*+*δ_I_*|/max|*δ_i_*,*δ_I_*| for *i* = (*X*,*Y*) and compared these *ε* values to a set threshold *ε_thr_*. We inferred that gene *X* masks *Y* when *ε_Y_*≤*ε_thr_* and *ε_X_*>*ε_thr_*; that gene *Y* masks *X* when *ε_X_*≤*ε_thr_* and *ε_Y_*>*ε_thr_*; or that the two genes are co-equivalent when *ε_Y_*≤*ε_thr_* and *ε_X_*≤*ε_thr_*. The mean relative standard error of all significant interaction terms (*δ_I_*≠0, t-test *p*-value <0.05) was used to define *ε_thr_* for a given trait dataset.

Influence parameters were calculated directly using experimentally measured trait values using the definitions in Table 3. Confidence intervals were obtained by estimating standard errors from replicate data.

## Supporting Information

Table S1 Comparison of measured and theoretical trait values for the eight different experimental conditions.
(DOC)

Table S2 Predicted values of the *β*-parameters in Eq. (1) given different assumptions.
(DOC)

Table S3 Signal-dependent interactions *δ_I_* and associated *p*-values obtained using fitness and expression traits.
(DOC)

Table S4 Influence parameters obtained from log2-transformed fitness data. The means (µ) and 95% confidence intervals (CI) of influences calculated from a minimum of four experimental replicate trait measurements are shown.
(DOC)

Table S5 Influence parameters obtained from log2-transformed expression data. The means (µ) and the 95% confidence intervals (CI) of influences calculated from a minimum of four experimental replicate trait measurements are shown.
(DOC)
