# Supplementary material for: Quantitative Epistasis Analysis and Pathway Inference from Genetic Interaction Data
Source: PLoS Comput Biol. 2011 May 12;7(5):e1002048. doi: 10.1371/journal.pcbi.1002048 (PMC3093353; doi:10.1371/journal.pcbi.1002048)
Supplement: Table S1 — Comparison of measured and theoretical trait values for the eight different experimental conditions. (DOC) [file pcbi.1002048.s001.doc]

**Table S1:** Comparison of measured and theoretical trait values for the eight different experimental conditions.

| **Positively regulated pathways** | | | | | | |
| --- | --- | --- | --- | --- | --- | --- |
| ***x*** | ***y*** | ***s*** | **Measured trait** | **Theoretical trait for**  **Pathways 1 & 2** | | **Theoretical trait for**  **Pathways 5 & 6** |
| 0 | 0 | 0 |  |  | |  |
| 1 | 0 | 0 |  |  | |  |
| 0 | 1 | 0 |  |  | |  |
| 1 | 1 | 0 |  |  | |  |
| 0 | 0 | 1 |  |  | |  |
| 1 | 0 | 1 |  |  | |  |
| 0 | 1 | 1 |  |  | |  |
| 1 | 1 | 1 |  |  | |  |
| **Negatively regulated pathways** | | | | | | |
| ***x*** | ***y*** | ***s*** | **Measured trait** | **Theoretical trait for**  **Pathways 3 & 4** | **Theoretical trait for**  **Pathways 7 & 8** | |
| 0 | 0 | 0 |  |  |  | |
| 1 | 0 | 0 |  |  |  | |
| 0 | 1 | 0 |  |  |  | |
| 1 | 1 | 0 |  |  |  | |
| 0 | 0 | 1 |  |  |  | |
| 1 | 0 | 1 |  |  |  | |
| 0 | 1 | 1 |  |  |  | |
| 1 | 1 | 1 |  |  |  | |

The pathway number refers to Table 1A in the main text.
