# Supplementary material for: Quantitative Epistasis Analysis and Pathway Inference from Genetic Interaction Data
Source: PLoS Comput Biol. 2011 May 12;7(5):e1002048. doi: 10.1371/journal.pcbi.1002048 (PMC3093353; doi:10.1371/journal.pcbi.1002048)
Supplement: Table S2 — Predicted values of the β-parameters in Eq. (1) given different assumptions. (DOC) [file pcbi.1002048.s002.doc]

**Table S2:** Isolating signal-specific gene deletion effects.

|  | | **(1)** | **(2)** | **(3)** |
| --- | --- | --- | --- | --- |
| **Pathway 1 & 2** |  |  |  | 0 |
|  |  |  |  | 0 |
|  |  |  |  | 0 |
|  |  |  |  |  |
|  |  |  | $-s_{Y}$ | $-s_{Y}$ |
|  |  |  |  |  |
| **Pathway 3 & 4** |  |  |  | 0 |
|  |  |  |  |  |
|  |  |  |  | 0 |
|  |  |  |  |  |
|  |  |  |  | 0 |
|  |  |  |  |  |
| **Pathway 5 & 6** |  |  |  |  |
|  |  |  |  |  |
|  |  |  |  |  |
|  |  |  |  | 0 |
|  |  |  |  | 0 |
|  |  |  |  | 0 |
| **Pathway 7 & 8** |  |  |  |  |
|  |  |  |  | 0 |
|  |  |  |  |  |
|  |  |  |  | 0 |
|  |  |  |  |  |
|  |  |  |  | 0 |

The table shows the predicted values of the *β*-parameters in Eq. (1) given different assumptions. The signal-specific effect of deleting the upstream gene is defined by *σ_XY_* = *σ_X_* +*σ_Y_* for pathways 1, 2, 5 and 6, and by *σ_XY_* = *σ_X_* - *σ_Y_* pathways 3, 4, 7 and 8. **Column (1):** No assumptions are made. **Column (2):** The magnitudes of signal-dependent gene influences (*σ_XY_*, *σ_Y_*) are much greater than the signal-independent influences (*α_X_* + *α_I_*; *α_Y_* + *α_I_*; and *α_I_*). **Column (3):** The signal-independent influences are negligible. The basal effect of deleting a gene that has signal-specific function always is always lower magnitude than the signal-specific effect for (2) and (3). It is also true when the basal and signal-specific effects have the same sign, i.e., when sign(*σ_XY_*) = sign(*α_I_* + *α_X_*); sign(*σ_Y_*) = sign(*α_I_* + *α_Y_*); and sign(*σ_Y_*) = sign(*α_I_*).
