# Supplementary material for: Quantitative Epistasis Analysis and Pathway Inference from Genetic Interaction Data
Source: PLoS Comput Biol. 2011 May 12;7(5):e1002048. doi: 10.1371/journal.pcbi.1002048 (PMC3093353; doi:10.1371/journal.pcbi.1002048)
Supplement: Table S3 — Signal-dependent interactions δI and associated p-values obtained using fitness and expression traits. (DOC) [file pcbi.1002048.s003.doc]

**Table S3:** Signal-dependent interactions *δ_I_* and associated *p*-values obtained using fitness and expression traits.

| Gene Pair | Fitness | | Expression | |
| --- | --- | --- | --- | --- |
|  | *δ_I_* | *p*-value | *δ_I_* | *p*-value |
| *gal1Δgal10Δ* | -3.13 | 1.80E-20 | -0.89 | 4.00E-05 |
| *gal1Δgal2Δ* | -1.55 | 1.30E-15 | -0.02 | 9.20E-01 |
| *gal1Δgal3Δ* | -1.53 | 2.80E-16 | 0.1 | 5.70E-01 |
| *gal1Δgal4Δ* | -1.52 | 1.80E-14 | 0.13 | 4.80E-01 |
| *gal1Δgal6Δ* | -0.85 | 2.60E-07 | 0.07 | 7.00E-01 |
| *gal1Δgal7Δ* | -3.8 | 1.80E-20 | -0.3 | 8.80E-02 |
| *gal1Δgal80Δ* | 0.36 | 2.10E-02 | -0.57 | 8.80E-04 |
| *gal10Δgal2Δ* | -2.96 | 1.20E-18 | -1.17 | 6.20E-07 |
| *gal10Δgal3Δ* | -2.94 | 3.70E-20 | -0.84 | 6.30E-05 |
| *gal10Δgal4Δ* | -3.01 | 2.20E-19 | -0.88 | 6.10E-05 |
| *gal10Δgal6Δ* | -0.47 | 2.50E-03 | -0.13 | 4.70E-01 |
| *gal10Δgal7Δ* | -3.59 | 2.60E-18 | -0.61 | 2.70E-03 |
| *gal10Δgal80Δ* | 0.28 | 1.20E-01 | -1.69 | 1.10E-10 |
| *gal2Δgal3Δ* | -0.13 | 1.30E-01 | -0.76 | 2.30E-04 |
| *gal2Δgal4Δ* | -0.16 | 7.70E-02 | -0.68 | 9.40E-04 |
| *gal2Δgal6Δ* | -0.49 | 1.30E-03 | -0.18 | 3.10E-01 |
| *gal2Δgal7Δ* | -1.15 | 7.80E-09 | -0.98 | 4.10E-07 |
| *gal2Δgal80Δ* | 1.26 | 2.50E-08 | -1.21 | 1.10E-08 |
| *gal3Δgal4Δ* | -0.39 | 2.50E-05 | -5.61 | 2.00E-21 |
| *gal3Δgal6Δ* | -0.46 | 8.10E-04 | 0.09 | 6.30E-01 |
| *gal3Δgal7Δ* | -3.61 | 2.00E-20 | -0.27 | 7.60E-02 |
| *gal3Δgal80Δ* | 0.09 | 5.60E-01 | -5.57 | 6.90E-23 |
| *gal4Δgal6Δ* | -0.5 | 5.60E-04 | 0.14 | 4.30E-01 |
| *gal4Δgal7Δ* | -3.72 | 1.90E-20 | -0.37 | 3.30E-02 |
| *gal4Δgal80Δ* | 1.89 | 3.00E-12 | -6.27 | 4.70E-22 |
| *gal6Δgal7Δ* | -0.44 | 9.50E-03 | 0.05 | 7.50E-01 |
| *gal6Δgal80Δ* | 0.23 | 2.10E-01 | -0.32 | 4.80E-02 |
| *gal7Δgal80Δ* | 0.85 | 8.40E-05 | -0.72 | 5.50E-06 |
