# Supplementary material for: Quantitative Epistasis Analysis and Pathway Inference from Genetic Interaction Data
Source: PLoS Comput Biol. 2011 May 12;7(5):e1002048. doi: 10.1371/journal.pcbi.1002048 (PMC3093353; doi:10.1371/journal.pcbi.1002048)
Supplement: Table S4 — Influence parameters obtained from log2-transformed fitness data. The means (µ) and 95% confidence intervals (CI) of influences calculated from a minimum of four experimental replicate trait measurements are shown. (DOC) [file pcbi.1002048.s004.doc]

**Table S4:** Influence parameters obtained from log2-transformed fitness data.

| Inferred Pathway | *α_X_* | | *α_Y_* | | *α_I_* | | *σ_S_* | | *σ_X_* | | *σ_Y_* | |
| --- | --- | --- | --- | --- | --- | --- | --- | --- | --- | --- | --- | --- |
|  | μ | CI | μ | CI | μ | CI | μ | CI | μ | CI | μ | CI |
| *S* → Gal1 → Gal10 → *T* | 0.004 | 0.066 | 0.038 | 0.047 | 0.095 | 0.091 | -1.687 | 0.105 | -1.589 | 0.152 | 2.962 | 0.24 |
| S → Gal2 → Gal1 → *T* | 0.175 | 0.081 | 0.002 | 0.079 | 0.097 | 0.097 | -0.454 | 0.07 | -1.399 | 0.047 | 1.539 | 0.128 |
| *S* → Gal3 → Gal1 → *T* | 0.249 | 0.033 | 0.103 | 0.026 | -0.004 | 0.062 | -0.52 | 0.085 | -1.332 | 0.053 | 1.539 | 0.128 |
| *S* → Gal4 → Gal1 → *T* | 0.188 | 0.076 | 0.123 | 0.077 | -0.024 | 0.093 | -0.683 | 0.19 | -1.169 | 0.171 | 1.539 | 0.128 |
| S → Gal1 → Gal7 → *T* | 0.208 | 0.098 | 0.093 | 0.101 | -0.11 | 0.128 | -1.67 | 0.14 | -2.262 | 0.14 | 3.618 | 0.119 |
| *S* → Gal2 → Gal10 → *T* | 0.253 | 0.09 | 0.115 | 0.131 | 0.018 | 0.124 | -0.469 | 0.173 | -2.807 | 0.196 | 2.962 | 0.24 |
| *S* → Gal3 → Gal10 → *T* | 0.232 | 0.076 | 0.12 | 0.029 | 0.013 | 0.103 | -0.525 | 0.06 | -2.751 | 0.18 | 2.962 | 0.24 |
| *S* → Gal4 → Gal10 → *T* | 0.305 | 0.053 | 0.273 | 0.079 | -0.14 | 0.076 | -0.62 | 0.167 | -2.656 | 0.266 | 2.962 | 0.24 |
| *S* → Gal10 → Gal6 → *T* | 0.09 | 0.074 | -0.1 | 0.083 | 0.043 | 0.115 | -3.266 | 0.045 | 2.496 | 0.213 | 0.456 | 0.258 |
| *S* → Gal10 → Gal7 → *T* | 0.162 | 0.056 | 0.012 | 0.085 | -0.029 | 0.117 | -3.3 | 0.185 | -0.632 | 0.131 | 3.618 | 0.119 |
| *S* → Gal2 → Gal6 → *T* | 0.408 | 0.085 | 0.079 | 0.094 | -0.136 | 0.105 | -0.435 | 0.16 | -0.334 | 0.149 | 0.456 | 0.258 |
| *S* → Gal3 → Gal4 → *T* | 0.156 | 0.04 | 0.076 | 0.025 | 0.089 | 0.059 | -0.471 | 0.046 | -0.195 | 0.055 | 0.352 | 0.135 |
| *S* → Gal3 → Gal6 → *T* | 0.417 | 0.09 | 0.115 | 0.023 | -0.172 | 0.089 | -0.502 | 0.049 | -0.267 | 0.238 | 0.456 | 0.258 |
| *S* → Gal3 → Gal7 → *T* | 0.283 | 0.083 | 0.021 | 0.08 | -0.038 | 0.113 | -0.516 | 0.056 | -3.416 | 0.183 | 3.618 | 0.119 |
| *S* → Gal4 → Gal6 → *T* | 0.337 | 0.069 | 0.115 | 0.06 | -0.172 | 0.089 | -0.62 | 0.137 | -0.149 | 0.134 | 0.456 | 0.258 |
| *S* → Gal4 → Gal7 → *T* | 0.309 | 0.093 | 0.128 | 0.08 | -0.145 | 0.086 | -0.56 | 0.118 | -3.372 | 0.258 | 3.618 | 0.119 |
| *S* → Gal7 → Gal6 → *T* | 0.154 | 0.076 | 0.114 | 0.022 | -0.171 | 0.079 | -3.948 | 0.109 | 3.178 | 0.295 | 0.456 | 0.258 |

The means (μ) and 95% confidence intervals (CI) of influences calculated from a minimum of four experimental replicate trait measurements are shown (see Table 3 for calculation).
