# Supplementary material for: Quantitative Epistasis Analysis and Pathway Inference from Genetic Interaction Data
Source: PLoS Comput Biol. 2011 May 12;7(5):e1002048. doi: 10.1371/journal.pcbi.1002048 (PMC3093353; doi:10.1371/journal.pcbi.1002048)
Supplement: Table S5 — Influence parameters obtained from log2-transformed expression data. The means (µ) and the 95% confidence intervals (CI) of influences calculated from a minimum of four experimental replicate trait measurements are shown. (DOC) [file pcbi.1002048.s005.doc]

**Table S5:** Influence parameters obtained from log2-transformed expression data.

| Inferred Pathway | *α_X_* | | *α_Y_* | | *α_I_* | | *σ_S_* | | *σ_X_* | | *σ_Y_* | |
| --- | --- | --- | --- | --- | --- | --- | --- | --- | --- | --- | --- | --- |
|  | μ | CI | μ | CI | μ | CI | μ | CI | μ | CI | μ | CI |
| *S* → Gal2 → Gal10 → *T* | -0.108 | 0.245 | 0.018 | 0.154 | 0.085 | 0.315 | 5.18 | 0.174 | -0.478 | 0.239 | 1.042 | 0.281 |
| *S* → Gal3 → Gal10 → *T* | -0.282 | 0.204 | -0.107 | 0.149 | 0.209 | 0.285 | -0.209 | 0.178 | 4.911 | 0.25 | 1.042 | 0.281 |
| *S* → Gal4 → Gal10 → *T* | -0.091 | 0.259 | -0.046 | 0.065 | 0.149 | 0.275 | -0.092 | 0.196 | 4.794 | 0.279 | 1.042 | 0.281 |
| *S* → Gal3 → Gal2 → *T* | -0.062 | 0.153 | -0.012 | 0.174 | -0.011 | 0.299 | 0.065 | 0.305 | 4.985 | 0.208 | 0.694 | 0.18 |
| *S* → Gal4 → Gal2 → *T* | 0.173 | 0.179 | 0.092 | 0.142 | -0.115 | 0.209 | 0.061 | 0.295 | 4.989 | 0.205 | 0.694 | 0.18 |
| *S* → Gal3 ↔ Gal4 → *T* | -0.202 | 0.184 | -0.071 | 0.124 | 0.129 | 0.178 | -0.064 | 0.243 | 0.136 | 0.165 | 5.672 | 0.16 |
| *S* → Gal3 *--|* Gal80-*-| T* | -0.182 | 0.128 | -0.433 | 0.203 | 0.108 | 0.189 | -0.79 | 0.197 | 0.182 | 0.065 | -6.352 | 0.149 |
| *S* → Gal4 → Gal7 → *T* | 0.056 | 0.142 | -0.018 | 0.084 | 0.001 | 0.157 | 0.09 | 0.257 | 5.301 | 0.289 | 0.353 | 0.185 |
| *S* -*-|* Gal80 -*-|* Gal4 → *T* | 0.043 | 0.061 | 0.426 | 0.193 | -0.368 | 0.176 | -0.008 | 0.36 | -0.08 | 0.246 | 5.672 | 0.16 |

The means (μ) and the 95% confidence intervals (CI) of influences calculated from a minimum of four experimental replicate trait measurements are shown (see Table 3 for calculation).
